# Intramedullary Nail Fixation Assisted by Orthopaedic Robot Navigation for Intertrochanteric Fractures in Elderly Patients

**DOI:** 10.1111/os.12447

**Published:** 2019-04-19

**Authors:** Hai Lan, Zhe Tan, Kai‐nan Li, Jin‐hua Gao, Tian‐hu Liu

**Affiliations:** ^1^ Department of Orthopaedics Affiliated Hospital of Chengdu University China; ^2^ Department of Orthopaedics The First People's Hospital of Anqing Anqing China; ^3^ Department of Orthopaedics Pidu District People's Hospital Chengdu

**Keywords:** Orthopaedic robot navigation, Intertrochanteric fracture, Intramedullary nail fixation

## Abstract

**Objective:**

To compare the clinical efficacy of intramedullary nail fixation for intertrochanteric fractures assisted by orthopaedic robot navigation and the traditional intramedullary nail fixation in elderly patients, and to investigate the application advantages of intramedullary nail fixation for femoral intertrochanteric fractures assisted by orthopaedic robot navigation in the elderly.

**Methods:**

Among the 51 patients with intertrochanteric fractures who were selected from April 2015 to September 2017 in the Affiliated Hospital of Chengdu University, 25 patients underwent the intramedullary nail fixation assisted by orthopaedic robot navigation (orthopaedic robot navigation surgery group) and 26 patients underwent the traditional intramedullary nail fixation (traditional surgery group). The operation time, the number of intraoperative fluoroscopy images taken, the frequency of guide pins inserted into the femoral marrow cavity, the amount of intraoperative bleeding, and the one‐time success rate of the guide pin inserted into the femoral marrow cavity were recorded. Fracture healing and internal fixation were observed. The Harris score was used to evaluate hip joint function 1 year after surgery.

**Results:**

All patients were followed up for 12–24 months. The operation time was 65.44 ± 8.01 min in the orthopaedic robot navigation surgery group and 77.50 ± 16.64 min in the traditional surgery group. The number of intraoperative fluoroscopy images taken was 10.28 ± 0.61 in the orthopaedic robot navigation surgery group and 13.23 ± 1.75 in the traditional surgery group. The frequency of guide pins inserted into the femoral marrow cavity was 1.00 ± 0.00 times in the orthopaedic robot navigation surgery group and 2.46 ± 1.10 times in the traditional surgery group. The one‐time success rate of intramedullary pin puncture was 100% (25/25) in the orthopaedic surgical robot navigation surgery group and 19.23% (5/26) in the traditional surgery group. The amount of surgical bleeding was 90.80 ± 14.98 mL in the orthopaedic robot navigation surgery group and 118.46 ± 32.21 mL in the traditional surgery group. Compared with the traditional surgery group, the operation time of the orthopaedic surgical robot navigation surgery group was shorter (*P* < 0.05), the number of intraoperative fluoroscopy images taken was fewer (*P* < 0.05), the frequency of guide pins inserted into the femoral marrow cavity was lower (*P* < 0.05), the one‐time success rate of intramedullary pin puncture was higher (*P* < 0.05), and the amount of surgical bleeding was less (*P* < 0.05). One year after surgery, fracture healing occurred in both groups without failure of internal fixation or fracture displacement. The Harris score of hip function in the orthopaedic robot navigation surgery group was 86.68 ± 6.23 and that in the traditional surgery group was 82.69 ± 6.85. It was higher than that in the traditional surgery group (*P* < 0.05). The fine rate of hip joint function in the orthopaedic robot navigation surgery group was 84.00% (21/25) and that in the traditional surgery group was 73.07% (19/26). There was no significant difference between the two groups (*P* > 0.05).

**Conclusion:**

Intramedullary nail fixation for intertrochanteric fractures assisted by orthopaedic robot navigation in elderly patients is an ideal method, offering a short operation time, minimal surgical trauma, less radiation, and good recovery of hip function.

## Introduction

Since the 1980s, with the development of medical science and technology and the increased level of difficulty and the precision required in surgical operations, there have been huge advances in surgical robotics. Robots was first used in brain surgery in 1985^1^. Surgical robots have the characteristics of good stability, flexible operation, precise movement, and good hand–eye coordination. They are being increasing used in clinical treatment. As the most typical representative of surgical robots^2^, the Leonardo Da Vinci surgical robot, a co‐development of Intuitive Surgical, International Business Machines Corporation (IBM), Massachusetts Institute of Technology (MIT), and Heartport in the United States, has been widely used in general surgery, thoracic surgery, urological surgery, obstetrics, and gynecological surgery. In 1992, surgical robots began to be used in orthopaedic surgery. Based on the current technical characteristics and application modes of orthopaedic surgical robots, they are mainly divided into haptic, autonomous, and passive types. They are commonly used in spinal surgery, joint replacement surgery, and orthopaedic trauma surgery for the hip and the pelvis. Outside China, orthopaedic surgical robots have developed rapidly, and the types of robots are relatively more comprehensive than those in China. The Robodoc robot system, the MAKO plasty robot system, the Acrobot robot system, and the Spine Assist robot system are commonly used in orthopaedic surgery in countries other than China. However, compared with the research and development of orthopaedic surgical robots abroad, the research and development of orthopaedic surgical robots domestically is still in its infancy in China. At present, there are two main types of orthopaedic surgical robots in China: remote control spine robots and navigation assistant robots. There are still many issues with the application of orthopaedic surgical robots, such as the exorbitant costs, the lack of a tactile sensory feedback system to prevent iatrogenic injury of patients, the relatively limited scope of application in orthopaedic surgery, and the large size of orthopaedic surgical robots. These are problems that clinicians and robot researchers need to solve urgently. In the future, the development direction of orthopaedic surgical robots will be miniaturization, specialization, and low cost of robots^3^. Nonetheless, with the development of minimally invasive surgery and the requirement of precise medical treatment, orthopaedic robot navigation assistance technology for orthopaedic surgery has been widely used in orthopaedic surgery due to its advantages in regard to safety, accuracy, and rapidity^4^. Navigation assistance technology is one of the core technologies of orthopaedic robots. Navigation can provide precise reference for the operation of robots. Navigation assistance technology uses the powerful data processing ability of computers to analyze and process the patient data acquired by medical image acquisition equipment (i.e. X‐ray and CT) for doctors to make surgical plans before and during surgery. At the same time, with the help of external space coordinate tracking equipment, the space–time coordinate measurement between the surgical instrument or robot and the target area of the patient is carried out, and the relative position relationship between the two is obtained, so as to guide the doctor to locate the target area of the patient accurately, quickly, and safely and insert the built‐in object^5^.

Intertrochanteric fractures account for nearly half of all hip fractures^6^. Coinciding with the aging of the population, the incidence of osteoporosis in the elderly has increased, and the incidence of intertrochanteric fractures has increased year by year^7^, ^8^. The treatment of intertrochanteric fractures of the femur can be divided into conservative treatment and surgical treatment. Due to the high incidence of conservative treatment complications, such as the need for bed rest and limb traction, the mortality rate within 1 year after injury is relatively high^9^, ^10^. Therefore, most scholars believe that surgical treatment should be the first choice for patients with femoral intertrochanteric fractures^11^. Surgical treatment is mainly divided into intramedullary fixation and extramedullary fixation. During intertrochanteric fracture intramedullary nail internal fixation, application of orthopaedic surgical robot navigation technology can help to accurately locate the puncture direction of the guide pin in intertrochanteric fractures, to improve the success rate of the one‐time guide pin inserted into the femoral marrow cavity, to shorten the operation time, to reduce the surgical trauma, and to facilitate the rapid postoperative rehabilitation of patients.

The purpose of this investigation is as follows: (i) to compare the clinical efficacy of intramedullary nail fixation for intertrochanteric fractures assisted by orthopaedic robot navigation and the traditional intramedullary nail fixation in elderly patients; (ii) to consider a new surgical method for intramedullary nail fixation for intertrochanteric fractures assisted by orthopaedic robot navigation in terms of the number of intraoperative fluoroscopy images taken, the frequency of guide pins inserted into the femoral marrow cavity, the one‐time success rate of intramedullary pin puncture, the fracture healing rate, and Harris score; and (iii) to discuss the superiority in application of this new surgical method.

## Materials and Methods

### *Inclusion and Exclusion Criteria*

#### *Inclusion Criteria*

Inclusion criteria were: (i) patients who were at least 65 years old with unilateral closed intertrochanteric fractures; (ii) patients who had undergone intramedullary nail fixation assisted by orthopaedic robot navigation or traditional intramedullary nail fixation; (iii) the main evaluation indicators include the operation time, the number of intraoperative fluoroscopy images taken, the frequency of guide pins inserted into the femoral marrow cavity, the amount of surgical bleeding, and the Harris score of hip function; and (iv) a retrospective case control study.

#### *Exclusion Criteria*

Exclusion criteria included: (i) pathological fracture (such as bone metastasis of cancer, primary bone tumor, and metabolic bone disease); (ii) history of fractures in the affected hip; (iii) bilateral femoral intertrochanteric fracture; (iv) the affected side of the hip suffers from moderate to severe arthritis or femoral head necrosis; and (v) postoperative follow‐up time was less than 1 year.

### *General Information of Participants*

Fifty‐one patients with intertrochanteric fracture undergoing intramedullary nail internal fixation in our department were selected from April 2015 to September 2017. According to the surgical method, the patients were divided into two groups for comparison, among which 25 patients underwent intramedullary nail fixation assisted by orthopaedic robot navigation and 26 patients underwent the traditional intramedullary nail internal fixation.

In the orthopaedic robot navigation surgery group, 25 patients, including 11 men and 14 women, were aged from 66 to 90 years, with an average age of 76.4 years. The patients were classified accordingly: 2 cases of type II, 10 cases of type III, and 13 cases of type IV.

In the traditional surgery group, 26 patients, including 14 men and 12 women, were aged from 65 to 88 years, with an average age of 76.5 years. The patients were classified according to Evans–Jensen type: 1 case of type I, 3 cases of type II, 13 cases of type III, and 9 cases of type IV.

Inner fixed implants used Zimmer Natural Nails Cephalomedullary Asia (ZNN), the proximal femoral anatomical intramedullary nails manufactured by Zimmer Biomet, Warsaw, Indiana, USA.

**Figure 1:**
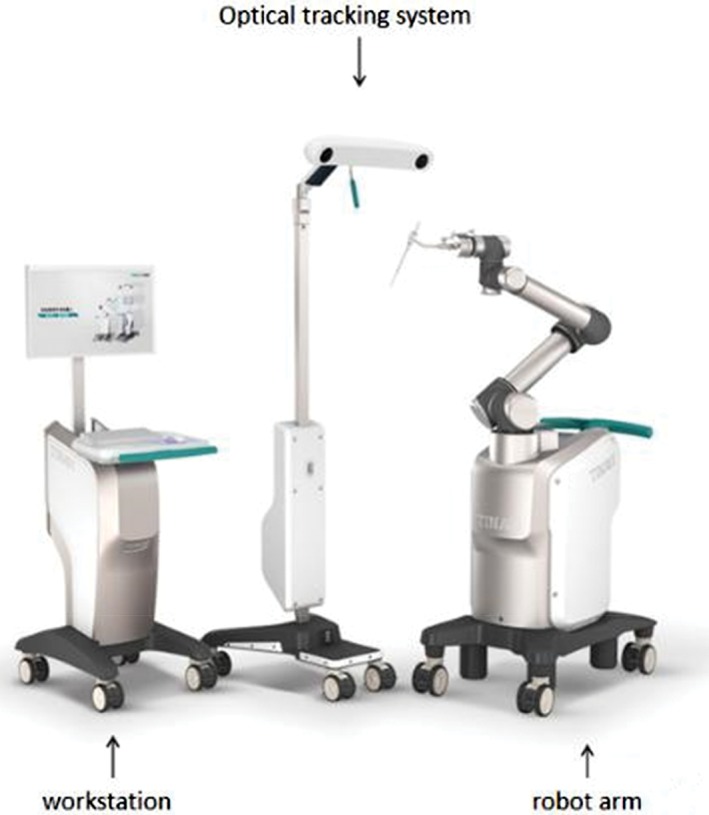
TiRobot, mainly composed of a workstation, an optical tracking system, and a robot arm (Photo provided by Beijing Tianzhihang Medical Technology).

In the orthopaedic robot navigation surgery group, the operation was performed with the help of TiRobot, the third generation of the orthopaedic surgical robot of Beijing Tianzhihang Medical Technology (Beijing, China) (Fig. 1).

### *Surgical Methods*

#### *Intramedullary Nail Fixation Assisted by Orthopaedic Robot Navigation*

##### (i) *Anesthesia and Position*

All patients in the group received surgery under general anesthesia. After successful anesthesia, the patient was placed in an orthopaedic traction bed with the pelvis in a horizontal position and the lower limbs of the healthy side fixed.

##### (ii) *Fracture Reduction and Traction*

The C‐arm X‐ray machine was assisted by fluoroscopy and the affected limb was treated with fracture manipulation reduction. After satisfactory fracture reduction, the affected limb received 15°continuous traction fixation.

##### (iii) *Robot Placement and Disinfection*

The operation was performed with the help of TiRobot. The orthopaedic surgical robot was prepositioned. The surgical area was routinely disinfected and covered with surgical towels. The positioning ruler and the robot arm were securely assembled. The positioning ruler was calibrated and adjusted to the appropriate position.

##### (iv) *Image Transmission*

**Figure 2:**
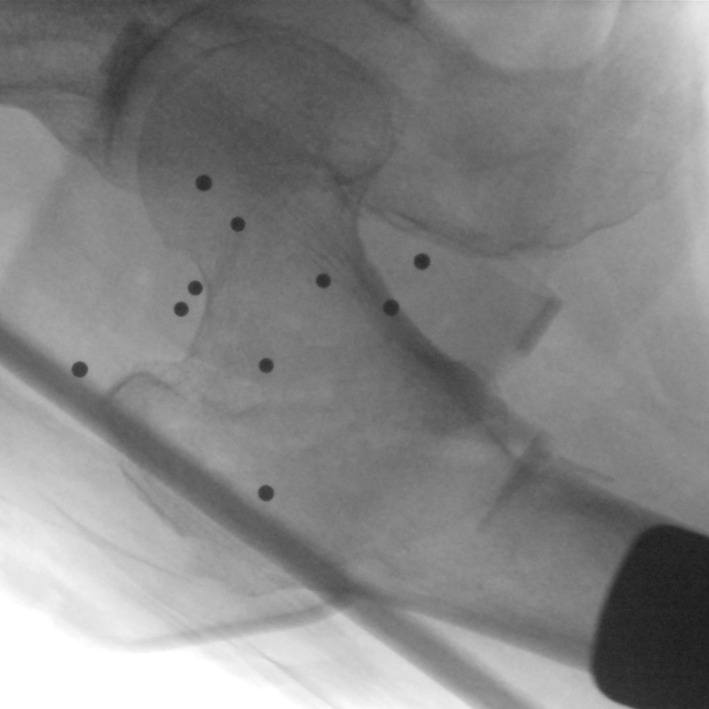
Anteroposterior image of the hip joint, with all 10 location points on the positioning ruler in it.

After the successful placement of the orthopaedic surgical robot, the C‐arm X‐ray machine was used to collect the anteroposterior and lateral images of the hip joint, respectively. Meanwhile, all 10 location points on the positioning ruler should be included in the X‐ray images (Fig. 2). The anteroposterior and lateral images collected by fluoroscopy using the C‐arm X‐ray machine were imported into the workstation.

##### (v) *Insertion Path Planning*

**Figure 3:**
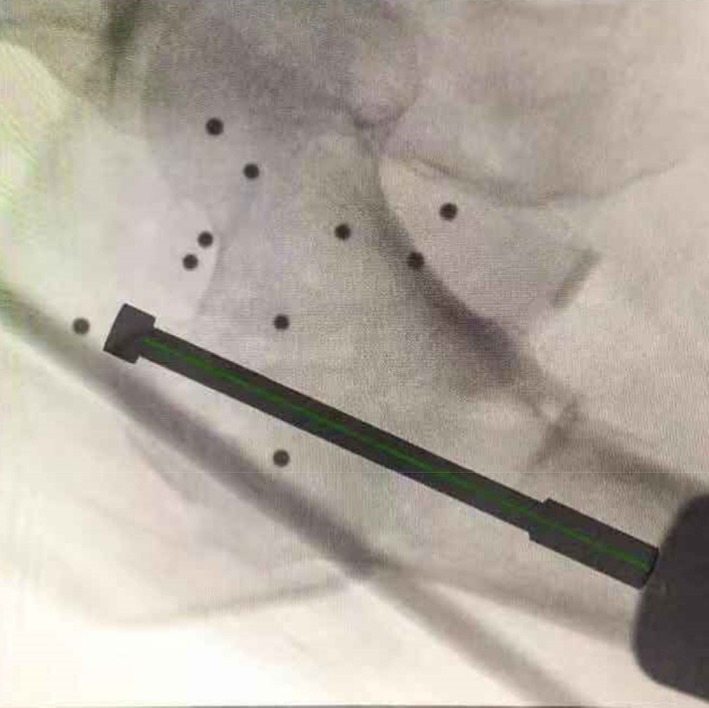
Anteroposterior image of the hip joint, which was imported into the workstation. Create a guide pin graph on the image which was imported workstation, adjust the position of the guide pin to plan the insertion path of the guide pin.

The insertion planning path and simulation figure of the guide pin were set in the workstation (Fig. 3).

##### (vi) *Robot Running*

**Figure 4:**
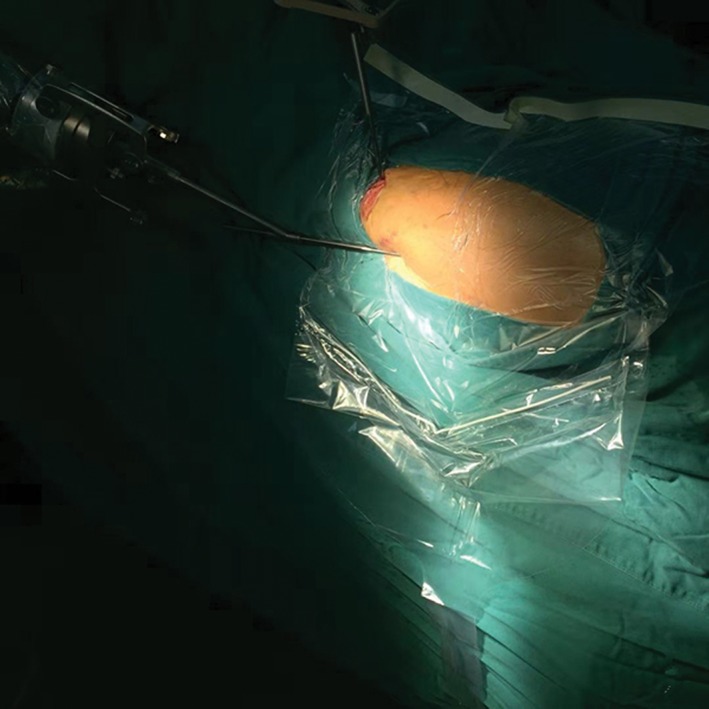
Photograph of the operative region of a patient’s hip. A guide pin was drilled into the femoral medullary cavity percutaneously assisted by the robot arm.

We ran the robot arm and drilled the guide pin into the femoral medullary cavity percutaneously, while the drilling direction and entry point of the guide pin were positioned according to the planned path navigation (Fig. 4).

##### (vii) *ZNN Insertion*

**Figure 5:**
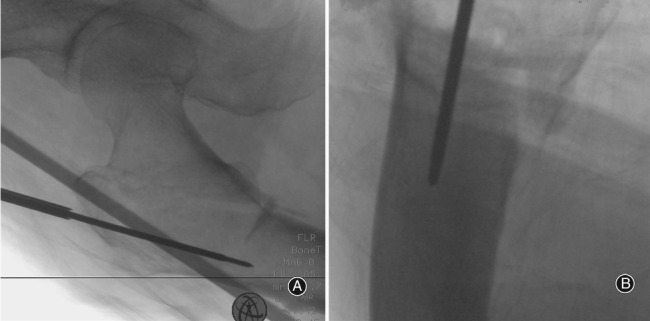
Anteroposterior and lateral images of the hip joint after insertion of the guide pin. The guide pin was confirmed to have penetrated the medullary cavity through the fractured part.

After an X‐ray was taken to confirm that the guide pin had penetrated the medullary cavity through the fractured part (Fig. 5), a horizontal 3‐4‐cm incision was made at the proximal point of the skin pin. After reaming the proximal pulp, the main screw was inserted, and the lag screw, the distal locking screw, and the nail cap were successively installed. Sutured the patient's operative incisions.

#### *Traditional Intramedullary Nail Fixation*

##### (i) *Anesthesia and Fracture Reduction*

All patients in the group had the same anesthesia, postural placement, and manual reduction standards as those in the orthopaedic robot navigation surgery group. A C‐arm X‐ray machine was used to view the hip joint and if the fracture reduction was satisfactory.

##### (ii) *Exposure*

We touched the apex of the greater trochanter from the outside of the skin and made a horizontal incision of 5–6 cm toward the proximal end. We separated the gluteus medius muscle and exposed the apex of the greater trochanter.

##### (iii) *ZNN Insertion*

We inserted the guide pin and took an X‐ray to determine whether the guide pin entered the medullary cavity. If the guide pin did not enter the medullary cavity, we pulled out the guide pin and inserted it again until the guide pin entered the medullary cavity. After reaming the proximal pulp, the main screw was inserted, and the lag screw, the distal locking screw, and the nail cap were successively installed. Sutured the patient's operative incisions.

### *Observation Indicators*

#### *Operation Time*

The operation time began after the sterile towel was laid and ended at the suture incision. It was mainly affected by the number of intraoperative fluoroscopy images taken. Repeated fluoroscopy during the operation will prolong the operation.

#### *Number of Intraoperative Fluoroscopy Images Taken*

We recorded the number of intraoperative fluoroscopy images taken during the operation, including each anteroposterior and lateral fluoroscopy image. Repeated insertion of guide pins into the femoral marrow cavity will increase the number of intraoperative fluoroscopy images taken.

#### *Frequency of Guide Pins Inserted into the Femoral Marrow Cavity*

The frequency of guide pins inserted into the femoral marrow cavity was recorded. Successful insertion of the main nail of ZNN needs to be guided by a guide pin. Whether the guide pin can successfully penetrate the broken end of the fracture into the femoral medullary cavity at one time will affect the length of the operation time and the number of intraoperative fluoroscopy images taken. The one‐time success rate of intramedullary pin puncture should be calculated after recording the frequency of guide pins inserted into the femoral marrow cavity.

#### *Amount of Surgical Bleeding*

The blood was collected by drainage bag. Prolongation of operation time increases the intraoperative blood loss.

#### *Fracture Healing*

All the patients were followed up and observed after surgery, and the orthotopic and lateral X‐ray images of the hip joint were reviewed at 1, 2, 3, 6, 9, and 12 months after surgery, respectively, to observe whether the implanted intramedullary nail was stable, the fracture line disappeared, and the fracture end was displaced. Patients were advised to return to the hospital for reexamination at least every 6 months 1 year after the surgery, to determine whether the internal fixation had failed.

#### *Harris Scoring*

The postoperative functional rehabilitation status was evaluated by Harris scoring. The hip function of all patients was evaluated 1 year after the operation. According to the Harris scoring standard^12^, ^13^, the pain, gait, and other functions of the affected hip joint, the degree of deformity and the results of physical examination of the joint activity were scored and graded. The clinical effects were: excellent, 90–100 points; good, 80–89 points; good, 70–79 points; poor, 70 points. We then calculated the fine rate of hip joint function.

### *Statistical Analysis*

Statistical software IBM SPSS 20.0 (International Business Machines Corporation, Armonk, New York, USA) was used for statistical analysis. The measurement data, including operation time, the number of intraoperative fluoroscopy images taken, frequency of guide pins inserted into the femoral marrow cavity, the amount of surgical bleeding, and the Harris score, were represented by mean ± standard deviation and were statistically analyzed using the *t*‐test. The data for the one‐time success rate of the guide pin inserted into the femoral marrow cavity was statistically analyzed with Fisher probabilities in a 2 × 2 table data test. This data, and the excellent and good rate of the Harris score, was statistically analyzed with the χ^2^‐test. *P* < 0.05 was considered statistically significant.

## Results

The intraoperative results are shown in Table 1.

**Table 1:** Comparison of the intraoperative information between the two groups (mean ± standard deviation)

	Number of cases	Operation time (min)	Number of intraoperative fluoroscopy images taken	Frequency of guide pins inserted into femoral marrow cavity	The amount of surgical bleeding (mL)
Orthopaedic robot navigation surgery group	25	65.44 ± 8.01	10.28 ± 0.61	1.00 ± 0.00	90.80 ± 14.98
Traditional surgery group	26	77.50 ± 16.64	13.23 ± 1.75	2.46 ± 1.10	118.46 ± 32.21
*t‐*value		3.277	7.968	6.618	3.906
*P‐*value		0.002*	<0.001*	<0.001*	<0.001*

* Statistically significant.

### *Operation Time*

The operation time was 65.44 ± 8.01 min in orthopaedic robot navigation surgery group and 77.50 ± 16.64 min in the traditional surgery group. Compared with the traditional surgery group, the operation time of the orthopaedic robot navigation surgery group was 15.56% shorter (*P* < 0.05).

### *Number of Intraoperative Fluoroscopy Images Taken*

The number of intraoperative fluoroscopy images taken was 10.28 ± 0.61 in the orthopaedic robot navigation surgery group and 13.23 ± 1.75 in the traditional surgery group. This was 22.30% lower in the orthopaedic robot navigation surgery group compared with the traditional surgery group (*P* < 0.05).

### *Frequency of Guide Pins Inserted into the Femoral Marrow Cavity*

The frequency of guide pins inserted into the femoral marrow cavity was 1.00 ± 0.00 times in the orthopaedic robot navigation surgery group and 2.46 ± 1.10 times in the traditional surgery group. This was 59.35% lower in the orthopaedic robot navigation surgery group compared with the traditional surgery group (*P* < 0.05). The one‐time success rate of intramedullary pin puncture was 100% (25/25) in the orthopaedic surgical robot navigation surgery group and 19.23% (5/26) in the traditional surgery group. The one‐time success rate of the guide pin inserted into the femoral marrow cavity was 4.20 times higher (*P* < 0.05). There were statistically significant differences between the two groups.

### *Amount of Surgical Bleeding*

The amount of surgical bleeding was 90.80 ± 14.98 mL in the orthopaedic robot navigation surgery group and 118.46 ± 32.21 mL in the traditional surgery group. This was 23.35% less in the orthopaedic robot navigation surgery group compared with the traditional surgery group (*P* < 0.05).

### *Fracture Healing Outcomes*

All patients were followed up for 12–24 months. All patients healed 1 year after surgery. The fracture healing rate was 100% (25/25) in the orthopaedic robot navigation surgery group and 100% (26/26) in the traditional surgery group, and no loosening of internal fixation or fracture displacement occurred.

### *Harris Score of Hip Function*

One year after the operation, the Harris score of hip function was determined in both groups. The Harris score of hip function in the orthopaedic robot navigation surgery group was 86.68 ± 6.23 and that in the traditional surgery group was 82.69 ± 6.85. The Harris score for the hip joint in the orthopaedic robot navigation surgery group was 4.83% higher than that in the traditional surgery group (*t* = 2.177, *P* = 0.034), and there was significant difference between the two groups.

According to the Harris score of the affected hip joint, the clinical efficacy of the patients in the orthopaedic robot navigation surgery group was excellent in 12 cases, good in 9 cases, fair in 4 cases, and excellent and good in 84.00% (21/25); the clinical efficacy of the patients in the traditional operation group was excellent in 6 cases, good in 13 cases, fair in 5 cases, poor in 2 cases, and excellent and good in 73.07% (19/26). There was no significant difference between the two groups (*χ* ^*2*^ = 0.368, *P* = 0.543).

## Discussion

In recent years, with the increasing number of patients undergoing intertrochanteric fracture surgery, the internal fixation of intertrochanteric fractures has been the focus of discussion. Intramedullary and extramedullary fixation have become the focus of attention. For femoral intertrochanteric fractures, especially unstable intertrochanteric fractures, intramedullary fixation has certain advantages over extramedullary fixation. Intramedullary therapy can increase the activity of patients and reduce the rate of surgical failure^14^, ^15^, ^16^. The intramedullary nail internal fixation is often affected by the experience of the surgeon. The degree of fracture reduction, unstable bare‐handed operation, and visual deviation make it difficult to ensure the success of a one‐time puncture when the guide pin is punctured. The guide pin often penetrates the medullary cavity from the broken end of the fracture. Repeatedly adjusting the guide pin puncture path will increase the number of punctures, which may cause re‐injury of muscles, soft tissues and bones, increase the degree of surgical trauma, and increase the amount of bleeding in patients. At the same time, prolonging the operation time, more fluoroscopy times, and increasing the exposure time of patients and medical staff to radiation will have a great impact on the health of both doctors and patients.

With the wider application and development of computer‐aided navigation in orthopaedic surgery, fracture internal fixation under the navigation of an orthopaedic surgical robot can obtain the best surgical path, the greatest surgical efficiency and accuracy, and the best surgical effect, with patients suffering less from surgical injury^17^. Orthopaedic surgical robots have precise navigation and positioning, and can plan the spatial positioning of surgical instruments. Through the movement of the manipulator arm, surgical instruments can be safely, accurately, and stably placed in the corresponding anatomical parts^18^, ^19^. Compared with manual operations, using an orthopaedic surgical robot ensures the consistency of the planning path and the surgical path. It can also monitor the movement of patients in real time and self‐correct the path. Orthopaedic surgical robots can precisely assist doctors in locating implants, and the accuracy can reach millimeter level. Minimally invasive surgery and high‐risk regional surgery under the guidance of orthopaedic surgical robot have obvious advantages, can reduce surgical complications, and can effectively reduce the risk of surgery.

The main results of this contrastive study can be summarized as follows. First, the operation time, the number of intraoperative fluoroscopy images taken, the frequency of guide pins inserted into the femoral marrow cavity, and the amount of surgical bleeding in the orthopaedic robot navigation surgery group were lower than those in traditional surgery group, and the difference was statistically significant (*P* < 0.05). Second, the one‐time success rate of the guide pin inserted into the femoral marrow cavity and the Harris score of hip function 1 year after surgery in the orthopaedic robot navigation surgery group were higher than those in the traditional surgery group, and the difference was statistically significant (*P* < 0.05). ZNN for intertrochanteric fractures assisted by orthopaedic robot navigation is a new operative method, and there have been, as yet, no relevant research reports. The results show that the new operative method can effectively reduce the operation time and the amount of bleeding, and facilitate early rehabilitation of patients. The Harris score for hip function is higher than that for the traditional operation, and the recovery of hip function of patients is better, which can significantly improve the curative effect.

Hollow screw internal fixation of intertrochanteric fractures of the femur under the navigation of an orthopaedic surgical robot simplifies the operation procedure. It can help the surgeon avoid inserting the guide pin repeatedly, making the operation more fluent. It can plan the path and position of the guide pin into the femoral medullary cavity through the platform of orthopaedic surgical robot planning. The manipulator can run independently and assist the surgeon to complete the placement of the guide pin. The precise positioning of orthopaedic robots helps surgeons to reduce the number of punctures during the operation, which increases the probability of successful insertion of a guide pin at one time. The traditional surgical method involves exposing the apex of the greater trochanter through incision before drilling the guide pin. Compared with the traditional operation, this method first cutaneous penetration into the femoral medullary cavity pin assisted by orthopaedic robot navigation. There is no need to make a long incision to expose the apex of the greater trochanter, just a small incision that allows the main nail to be inserted into the medullary cavity. And there is no excessive stripping of the gluteus medius muscle, which reduces the incidence of hip abduction muscle strength decline after surgery. Therefore, it effectively reduces the amount of bleeding and the degree of surgical trauma suffered by patients, makes the operation safer and more effective, and is conducive to the healing of fractures and early rehabilitation exercises after surgery. This method also reduces the number of intraoperative fluoroscopy images, reduces the damage caused by X‐ray radiation to both doctors and patients, and plays a protective role for patients and medical staff.

To sum up, intramedullary nail fixation for intertrochanteric fractures assisted by orthopaedic robot navigation in elderly patients is an ideal method, offering a short operation time, minimal surgical trauma, less radiation, less bleeding, a higher one‐time success rate of the guide pin inserted into the femoral marrow cavity, and good recovery of hip function. However, there are still some shortcomings in this study: the number of cases collected is relatively small and the follow‐up time of some patients is relatively short. In the future, the number of studies should be increased and the follow‐up time of patients should be longer.
